# Editorial: Pathological fatigue from neurons to behavior

**DOI:** 10.3389/fnbeh.2022.973190

**Published:** 2022-07-28

**Authors:** Birgitta Johansson, Ekaterina Dobryakova, Joukje van der Naalt

**Affiliations:** ^1^Sahlgrenska Academy, University of Gothenburg, Gothenburg, Sweden; ^2^Kessler Foundation, West Orange, NJ, United States; ^3^Department of Neurology, University Medical Center Groningen, Groningen, Netherlands

**Keywords:** pathological fatigue, neurological disorder, hypothesis, brain function, work ability, behavior, treatment

Pathological fatigue is one of the most distressing and long-lasting symptoms following neurological disorders and has a considerable negative impact on quality of life and work ability. Despite this, pathological fatigue is not well understood and acknowledged. While research has been scarce on this issue, despite increasing attention during the last 10 years, important knowledge gaps need to be explored. The aim of the current Research Topic was to collect promising, recent, and novel research output dissecting and advancing our knowledge on pathological brain fatigue from neurons to behavior.

A hypothetical explanation to pathological fatigue is presented by Rönnbäck and Johansson, suggesting a dysfunction in the astroglial support of the glutamate transmission. The astroglial cells are responsible for the fine-tuning of the glutamate transmission, but this capacity is attenuated and associated with neuro-inflammation. This enhances astrocyte swelling and impaired glutamate uptake followed by a decrease in extracellular space, with glutamate diffusion to adjacent neurons. This process would result in decreased precision in glutamate signaling, probably more unspecific and more energy demanding. Attenuation of glutamate transmission will also reduce GABA and might reduce information filtering in the brain, potentially providing an explanation to the feeling of being overwhelmed by information. In addition, glucose uptake by the astrocytes may be decreased. Reduced glucose availability in many brain regions could account for reduced dopamine, noradrenaline, serotonin, and acetylcholine signaling. Thus, this hypothesis could explain the reduced ability to perform cognitive tasks over longer periods of time by a deficiency in energy supply of the neuronal network.

The neurological underpinning of fatigue was explored in several patient groups. One of the most persistent and debilitating symptoms of Multiple Sclerosis (MS) is cognitive fatigue. Román et al. investigated the Signal Detection Theory (SDT) and the relationship between cognitive fatigue and brain activation. The results showed that the SDT metric of Criterion (which measures the ability to discriminate a target or not) was positively correlated with subjective cognitive fatigue. The MS group became more conservative in their response pattern compared to controls as fatigue increased, and required more evidence about whether something was a correct or incorrect before responding during the working memory task. Moreover, the activation in brain areas previously shown to be related to cognitive fatigue, such as the striatum, was also related to Criterion. It was suggested that the metrics of SDT may represent a novel tool with which to study cognitive fatigue in MS and other neurological populations.

In the paper by Ramage et al., participants suffering from mild traumatic brain injury (TBI) and an orthopedic control group performed an alternating effort task to examine sustained attention. Brain connectivity was analyzed using functional MRI data. The mild TBI participants showed elevated cingulo-opercular connectivity (CO) irrespective of task demand, while the connectivity in the orthopedic control group decreased over time. Fronto-parietal (FP) connectivity was lower in the mild TBI group. Hyperconnectivity of CO along with hypoconnectivity of the FP may allow for attaining of tasks but also contributes to fatigue.

Chronic fatigue is also a prominent symptom in many sarcoidosis patients, affecting quality of life and interfering with treatment. Kettenbach et al. included in their study patients with a histological diagnosis of sarcoidosis, with two-thirds of them suffering from chronic fatigue. Chronic fatigue patients showed more symptoms of depression and anxiety, and lower quality of life. During a working memory task measuring brain activity with functional MRI, those suffering from chronic fatigue showed a smaller increase in brain activation with increasing task difficulty vs. the group without fatigue. It was concluded that inadequate adjustment of brain activation with increasing demands can be a potential neurobiological marker of chronic fatigue in sarcoidosis patients.

Fatigue after aneurysmal subarachnoid hemorrhage (aSAH) is frequent many years after the insult. Western et al. reported fatigue to be associated with depression, with incomplete overlap as not all were characterized as depressed but suffered from fatigue, indicating fatigue and depression to be distinct entities. Fatigue was not related to neurological status and cognitive functioning using 24 standard neuropsychological tests, but was related to quality of life and return to work.

Johansson investigated a screening method for fatigue, using the Work Ability Index (WAI) and Mental Fatigue Scale (MFS). Participants commonly affected by mental fatigue, acquired brain injury (traumatic brain injury, stroke), burn-out syndrome and hypothyroidism were included. The results showed that there was a significant relationship between WAI and MFS and both were significant predictors for work status. It was concluded that mental fatigue is related to work ability, and WAI and MFS are suggested to be used for screening of work ability. This may also be useful for other groups of patients who commonly suffer from mental fatigue. Identifying mental fatigue can help people receive rehabilitation and promote a sustainable and well-functioning workplace and wellbeing of the individual.

Connolly et al. aimed to invest a novel in-home light therapy for fatigue and sleep disturbance in individuals with TBI or stroke, all suffering from fatigue. The clinical trial did not show a statistically significant benefit on fatigue.

In this topic several patient groups were included, all suffering from long term pathological fatigue. A hypothetical explanation to pathological fatigue was presented, and connection between pathological fatigue and brain functioning was explored by different diagnostic methods to measure cognitive function, quality of life and work ability in several patient groups. More effort is needed to develop efficient treatment methods. It is important to focus research efforts to reduce the existing gap between neuronal functioning and behavioral outcomes in relation to the pathological condition and symptoms.

## Author contributions

All authors listed have made a substantial, direct, and intellectual contribution to the work and approved it for publication.

## Conflict of interest

The authors declare that the research was conducted in the absence of any commercial or financial relationships that could be construed as a potential conflict of interest.

## Publisher's note

All claims expressed in this article are solely those of the authors and do not necessarily represent those of their affiliated organizations, or those of the publisher, the editors and the reviewers. Any product that may be evaluated in this article, or claim that may be made by its manufacturer, is not guaranteed or endorsed by the publisher.

